# Analysis of muscle synergy in the lower extremities and associated adaptive strategies during single-leg landing

**DOI:** 10.3389/fphys.2025.1496274

**Published:** 2025-07-25

**Authors:** Heyu Fu, Dan Yu, Yaru Chen, Wenxiao He, Yiting Duan, Fan Gao, Haibin Liu

**Affiliations:** ^1^ Institute of Physical Education and Health, Department of Physical Education, Dalian University of Technology, Dalian, China; ^2^ Liaoning Key Lab of IC and BME System, The School of Biomedical Engineering, Dalian University of Technology, Dalian, Liaoning, China; ^3^ College of Education, Department of Kinesiology and Health Promotion, University of Kentucky, Lexington, KY, United States

**Keywords:** single-leg descent, muscle synergy, OpenSim simulation, landing process, biomechanical simulation

## Abstract

**Objective:**

This study aims to utilize OpenSim simulation technology to explore the muscle synergy in the lower extremities during single-leg landing and associated adaptive trategies.

**Methods:**

Twelve participants were recruited to perform single-leg landing tasks from various heights and distances. Kinematic and kinetic data were collected using the Vicon motion capture system, AMTI force platforms, and Noraxon electromyography system. Joint angles and muscle activations were computed using OpenSim.

**Results:**

The number of muscle synergy modules and the Variance Accounted For values showed high consistency across participants. Three muscle synergy modules were identified for landing tasks performed at 30 cm height with 0 cm and 30 cm horizontal distances, and at 45 cm height with 50 cm horizontal distance. Four modules were found for tasks performed at 30 cm height with 50 cm horizontal distance, and at 45 cm height with 0cm and 30 cm horizontal distances. The structure and activation timing of muscle synergy modules varied with changes in landing height and horizontal distance. Notably, the hip flexion angle significantly increased during the landing task at 30 cm height with 50 cm horizontal distance; the peak angles of the knee and ankle joints significantly increased at 45 cm height with 50 cm horizontal distance.

**Conclusion:**

The study demonstrates that structures and activation of muscle synergy vary with changes in landing height and horizontal distance, while showing high similarity in muscle synergy outcomes among participants. Moreover, landing height significantly affects the knee and ankle joints, while horizontal distance significantly influences the knee and hip joints.

## 1 Introduction

Single-leg landing refers to the action where only one leg makes contact with the ground and supports the body (Kunugi et al., 2020). This movement pattern is commonly observed in daily activities and sports, such as basketball rebounding and volleyball spiking (Tai W et al., 2019; Xu D et al., 2021). Improper techniques and overloading may lead to various types and severities of sports injuries (Zhang et al., 2014). For example, the high impact force during landing can increase muscular activation in the lower limb joints, potentially resulting in injuries such as ligament tears, fractures, and bone contusions (Yeow et al., 2008).

The human body employs various landing strategies during single-leg descents. Several studies have investigated the motor performance of single-leg landings from different vertical heights (Pappas et al., 2007; Elf and Paine, 2001). It has been observed that as falling height increases, biomechanical parameters such as the flexion and extension angles of the lower limb joints, the peak ground reaction force and the time to its occurrence change significantly. Specifically, the peak ground reaction force increases, its occurrence time becomes earlier, and the peak flexion and extension angles of the hip, knee, and ankle joints, as well as their corresponding timing, gradually prolong (Wang et al., 2017). In addition, the orientation of single-leg landings (e.g., forward, 45° lateral, and medial) significantly affects foot and ankle kinematics and dynamic postural stability. Among these, 45° lateral landings pose a higher risk of stretching the lateral ligament of the ankle (Kunugi et al., 2020). Furthermore, lateral and diagonal jump landings tend to produce higher medial/lateral stability indices, while forward jump landings yield higher vertical stability indices (Wikstrom et al., 2008). These biomechanical changes place greater demands on the synergistic activity of lower extremity muscles, influencing joint stability and increasing injury risk (Lin et al., 2023). However, direct methods for exploring the state of musculoskeletal movement and its injury prevention mechanisms remain elusive.

In recent years, biomechanics has become a widely adopted approach for analyzing movement patterns during single-leg landings. Hovey S et al. (2019) conducted a study using 3D motion capture systems and force plates to examine lower limb kinematics, dynamics, and gender differences among athletes during single-leg landings, drop jumps, and countermovement jumps. Similarly, Pang and Tsang, (2022) employed the Vicon system to measure the angular maxima of the hip, knee, and ankle joints in the single-leg lateral landing tests, demonstrating the reliability of three-dimensional motion analysis following anterior cruciate ligament reconstruction. With the advancement of computer simulation technology, researchers have utilized OpenSim to investigate the biomechanics of kinematics and kinetics during single-leg landings, focusing particularly on forces around the knee joint during the single-leg support phase (Morgan K D et al., 2014). Maniar et al. (2020) studied the adaptation of lower limb muscles to complex loads on the knee joint during single-leg landings, emphasizing critical biomechanical factors such as tibiofemoral shear forces, knee valgus moments, and rotational torques. Moreover, Mokhtarzadeh et al. (2014) compared static optimization algorithms and muscle computation control methods for predicting muscle forces during single-leg landings, validating the accuracy of both approaches using empirical data.

Many studies employing 3D motion capture and simulation techniques have primarily focused on the biomechanical aspects of single-leg landings, investigating the kinematics and dynamics of the lower limbs. Though these studies offer a valuable scientific basis for sports training and injury prevention, they provide limited insight into neuromuscular control mechanisms. Muscle synergies are neural modules through which the central nervous system simplifies movement control by coordinating groups of muscles into low-dimensional, task-specific activation patterns (Bizzi and Cheung, 2013). This combination of muscles, known as muscle synergy, simplifies the control of motion. By modulating the activation patterns of these synergies, researchers can describe neural control strategies, elucidate muscle coordination mecanicsms (Zhang et al., 2021), improve the learning of correct technical movements, and reduce the risk of sports injuries (Hirashima and Oya, 2015). There is a growing trend among scholars to integrate biomechanical analysis with motor control theory, investigating variations in muscle activation strategies, synergy modules, and temporal patterns across diverse movements (Chen et al., 2023; Hagedoorn et al., 2022; Park and Caldwell, 2022). This simulation approach not only enables cross-validation of the recorded surface electromyography (sEMG) signals but also provides estimates of deeper muscle activations that are not accessible through surface electrodes. Given that traditional sEMG, while noninvasive and easy to operate, typically cannot capture the activity of small, deep muscles crucial for posture and balance—and that intramuscular EMG, despite its high spatial resolution, is too invasive for large-scale or long-term studies—the integration of simulation data effectively overcomes these limitations and yields a more comprehensive characterization of muscle recruitment patterns and neuromuscular control dynamics (Seth et al., 2018; Chen et al., 2024).

However, most existing studies focus on movements involving large muscle groups, such as walking, running, and crawling, with a particular emphasis on movement disorders such as stroke and spinal cord injury (Van Criekinge et al., 2020). In contrast, research on the motion control mechanism underlying single-leg landing is limited, and currently there is no systematic analysis of how the neuromuscular system regulates these movements. A well-developed computational model of muscle synergy holds the potential to significantly advance sports researchers’ understanding of how the central nervous system regulates and coordinates muscle activity through muscle synergies to perform complex motor tasks. This concept is foundational in both biology and neuroscience (Zhang et al., 2021). In summary, a comprehensive and in-depth investigation into the biomechanical parameters of the lower limbs and the role of muscle synergies during single-leg landings under varying conditions is crucial for revealing movement strategies involved in these tasks.

In the field of sports science and injury prevention, understanding the biomechanical characteristics of lower limbs landings is essential for informing training strategies and mitigating injury risks in athletes. Previous researches have shed light on the kinematic and dynamics of bilateral landings, revealing how various factors influence stability and muscle activity (Niu et al., 2013). For example, Niu et al. (2013) evaluated dynamic postural stability and muscle activity during bilateral landings. Sixteen participants (8 male and 8 female) performed landings from heights of 32 cm, 52 cm, and 72 cm. The results indicated that as landing height increased, horizontal time to stabilization significantly decreased, and lower extremity muscle activity enhanced, while vertical time to stabilization remained unchanged. Although bilateral landings generally exhibit stable postures, the body becomes more vulnerable to injury before stability is fully restored. This suggests that athletes may prioritize tissue protection over dynamic stability in training. Similarly, Wang and Peng (2013) compared the biomechanical characteristics of unilateral and bilateral drop jumps in male college students from heights of 20 cm, 30 cm, 40 cm, and 50 cm. The findings showed that as landing height increased, the peak impact force during unilateral drops rose significantly, with 30 cm identified as the optimal height for balancing jump performance and joint stiffness. Moreover, unilateral drops from 30 cm exhibited superior impact and propulsion forces compared to other heights. These observations highlight that different landing heights impose varying biomechanical demands on the lower limbs, with greater heights placing increased loads on the body.

Muscles, bones, and soft tissues collectively facilitate movement, with research on muscle coordination primarily focusing on activation patterns. In rehabilitation, studying muscle coordination helps to understand both normal and abnormal muscle operation patterns, develop more effective rehabilitation plans, and guide patients in rebuilding normal movement patterns. Previous studies have mainly concentrated on level-ground or bilateral support movements. In contrast, unilateral landings are more prone to causing injuries in military, sports, and everyday activities, increasing the risk of falls and involving more complex injury mechanisms. Therefore, this study investigates muscle coordination during unilateral landings from different heights, providing data and insights for injury prevention.

This study aims to investigate the neuromuscular control mechanisms involved during single leg landing by imploying OpenSim simulation technology to analyze lower limb muscle synergy, joint angles, muscle activations, and muscle synergy patterns. First, kinematic, kinetic data, and electromyographic data were collected during single leg landing tasks under varying height and horizontal distance conditions by using Vicon motion capture systems, 3D force platforms, and Noraxon System. Then,we integrates muscle activation data from sEMG with simulation techniques, non-negative matrix factorization algorithms (Rabbi et al., 2020) were applied to uncover the intrinsic patterns of muscle synergy. This approach provides valuable insights into the regulatory mechanisms governing muscle synergy contraction during single-leg landings. The findings offer a detailed understanding of the role of neuromuscular systems in complex movement control, enriching the theoretical foundations of biomechanics and shedding new light on muscle coordination mechanisms during movement execution.

## 2 Methods

### 2.1 Participant

Twelve healthy male subjects (age: 22.7 ± 2.4 years; height: 179.3 ± 4.6 cm; weight: 71.8 ± 7.2 kg) from Dalian University of Technology volunteered to participate in this study. None of the subjects reported lower limb injuries. Moreover, all of them were right-limb dominant, as indicated by their preference of starting to walk with their right foot. Before the experiment, all participants were informed about the study’s purpose, procedures, and potential risks before providing their written informed consent to participate. All procedures were conducted in compliance with the Declaration of Helsinki on medical research involving human subjects.

### 2.2 Experimental design

The subjects wore 39 reflective markers for static and dynamic motion tracking (as shown in Figure 2a), which were captured by an eight-camera Vicon system at a sampling frequency of 200 Hz (Vicon, Oxford Metrics Limited, UK). Synchronously, the ground reaction force and moments were recorded by using a force platform at 1,000 Hz (AMTI, Watertown, United States) during single-leg landing. Eight-channel sEMGs were recorded using the Noraxon System at 2000 Hz (Noraxon Ultium EMG, United States) from the muscles in the right leg: Rectus Femoris (Rect_fem), Vastus Lateralis (Vas_lat), Vastus Medialis (Vas_med), Biceps Femoris (Bi_fe), Tibialis Anterior (Tibant), gastrocnemius medialis (Gas_med), gastrocnemius lateralis (Gas_lat), Soleus (Sol).

**FIGURE 1:**
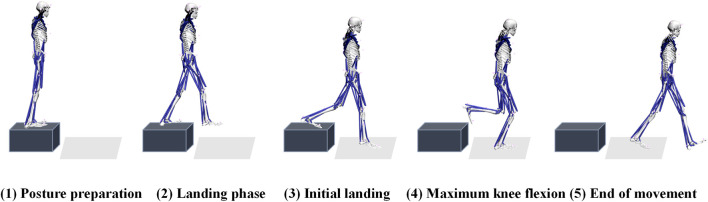
Schematic diagram of experimental movement and preparation. The figure presents the experimentally captured process of a single-leg drop motion, delineating five characteristic phases: (1) Posture Preparation, (2) Landing Phase, (3) Initial Landing, (4) Maximum Knee Flexion, and (5) End of Movement.

On the day of the experiment, participants began with a warm-up session and acclimatized to the laboratory environment. They performed single-leg landing maneuvers from various heights (30 cm and 45 cm) and horizontal distances (0 cm, 30 cm, and 50 cm) on a platform. The selected heights represent a gradient of impact loads, with 30 cm indicating moderate loading and 45 cm reflecting higher mechanical demands. Similarly, the horizontal distances range from purely vertical landings (0 cm) to those requiring increased forward motion (50 cm), representing a progression from low to high neuromuscular and biomechanical challenges (Yeow et al., 2009; Ali et al., 2012; Niu et al., 2011). As shown in Figure 1, each landing was performed with only the right leg making contact with the force platform. The horizontal distance from the edge of the platform to the inside edge of the force platform was defined as “Length (L)”, and the landing height was defined as “Height (H)”. Each experimental condition was separated by a 30-s interval, and participants repeated the single-leg landing maneuver three times under each condition. This resulted in a total of 2 × 3 × 3 sets of experimental data collected per participant. Throughout the test, participants were instructed to keep their arms raised to shoulder height. The complete cycle of single-leg landing was defined as the period from when the right leg made contact with the force plate until it completely left the force plate.

### 2.3 Biomechanical simulation

**FIGURE 2:**
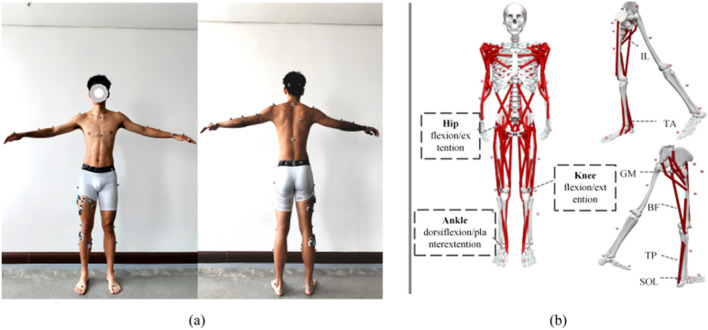
Experimental setup. **(a)** Illustrates the acquisition of full-body images with 39 marker points during motion capture, establishing the “Plug-gait Full Body Model” and the placement of 8 EMG channels for the collection of EMG data. **(b)** Demonstrates the “Arm Swing Model” in OpenSim (Goudriaan et al., 2014).

This study employed the “Arm_Swing” whole-body model (Goudriaan et al., 2014) based on OpenSim software, which consists of 102 muscles, 32 joints and 57 degrees of freedom. The model was aligned with 39 marker points individually, as shown in Figure 2b. An individualized model for each participant was created by scaling the generic model according to the measured height, weight, and other joint size parameters. The motion data was then analyzed using the inverse kinematics algorithm to derive changes in joint angles over time. By integrating motion data with biomechanical parameters, the study computed changes in joint torques and estimated muscle activation levels and forces required to achieve the observed motion states through static optimization methods (Delp et al., 2007; Seth et al., 2018).

### 2.4 Muscle synergy analysis

The sEMG signal processing procedure (De Luca et al., 2010) involves several steps. The preprocessing steps included notch filtering, high-pass filtering, full-wave rectification, low-pass filtering, and amplitude normalization. Specifically, a notch filter was applied to remove 50–60 Hz power line interference and to retain only the effective frequency range of 20–500 Hz. To eliminate low-frequency motion artifacts caused by electrode shift or skin movement, a fourth-order Butterworth high-pass filter with a 30 Hz cutoff was used. The filtered signals were then full-wave rectified to convert the bipolar sEMG into unipolar signals, allowing for more accurate estimation of muscle activation levels. Subsequently, a zero-phase fourth-order Butterworth low-pass filter with a cutoff frequency of 5 Hz was applied to generate the EMG linear envelope, approximating the low-pass characteristics of muscle force generation. Finally, the EMG signals were normalized to the peak amplitude observed within the entire movement cycle for each muscle, allowing for inter-muscle and inter-subject comparisons. This preprocessing allows for comparison with muscle activations derived from the OpenSim simulation.

This preprocessing allows for comparison with muscle activations derived from the OpenSim simulation. Muscle synergies were extracted from sEMG envelope by utilizing the Non-Negative Matrix Factorization (NNMF) algorithm. In the analysis of muscle synergies, the original surface electromyography (sEMG) envelope signals matrix M, with dimensions X*T, is decomposed into a linear combination of muscle synergy weight matrices W, and time-dependent activation coefficient matrices C, as described by the Equations 1, 2 below:

$$M_{X\times T}≅W_{X\times S}\times C_{S\times T}+E$$ (1)

where E represents the residual matrix, with smaller values indicating a better matrix decomposition and lower error. M denotes the sEMG envelope matrix, X represents the number of muscle synergy groups, and T is the length of the sample time series, that is, the number of sampling points. W is the muscle synergy structure matrix with dimensions X*S, and C is the muscle synergy activation coefficient matrix with dimensions S*T. After applying NMF, the optimal number of muscle synergies S is determined by evaluating the VAF of the reconstructed original EMG matrix. The VAF quantifies the proportion of the variance in the original data that is accounted for by the muscle synergy model, providing an index of the model’s explanatory power. A higher VAF value indicates a better fit of the muscle synergy model to the data, suggesting a more accurate representation of the underlying muscle activation patterns (Lee and Seung, 1999; Yang et al., 2020). In this study, a threshold of VAF ≥0.9 was selected, ensuring that the muscle synergy modules have high reconstruction accuracy, good stability, and strong physiological significance (Rabbi et al., 2020). The relationship between reconstruction accuracy and the number of muscle synergies is expressed by the following equation:

$$VAF=1-\frac{\sum_{i=1}^{X}\sum_{j=1}^{T}{\left(M-M_{con}\right)}^{2}}{\sum_{i=1}^{X}\sum_{j=1}^{T}{\left(M-\bar{M}\right)}^{2}}$$ (2)

where X and T represent the number of muscles examined and the number of sampling points, respectively. M is the sEMG envelope matrix to be decomposed, and M_con_ is the constructed matrix, which varies according to different muscle synergy models (Allen et al., 2019).

### 2.5 Selection of indicators and statistical analysis

Firstly, the normality of these datasets was tested by employing the Shapiro–Wilk test. Then, a two-way ANOVA was conducted to test whether there were significant differences in the maximum peak angles of hip joint flexion, knee joint flexion, and ankle plantar flexion across different jump heights and horizontal distances. In addition, the similarity between muscle synergies was analyzed by calculating Pearson’s Correlation Coefficient. All statistical analyses were performed using the SPSS version 21 software (IBM Corp., Armonk, NY, United States), and the results of the experiments were represented as mean ± standard deviation (Mean ± SD), with significance level set as *P* < 0.05.

## 3 Result

### 3.1 Accuracy verification for results from skeletal muscle simulation

**FIGURE 3:**
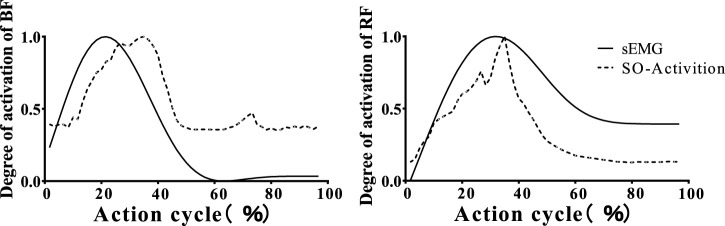
OpenSim static optimization of muscle activation and sEMG signal comparison. The figure compares the EMG signals (solid line) collected from the biceps femoris and rectus femoris muscles with the muscle activations (dashed line) calculated by OpenSim.

To compare and analyze the muscle activation levels computed by the OpenSim optimization algorithm, we obtained the muscle activation data after EMG acquisition and processing. This included the right leg muscles: GM, IL, BF, RF, GAST, TA, TP and Sol, with activation values ranging from 0 (fully deactivated) to 1 (fully activated). As shown in Figure 3, the muscle activation levels of BF and RF throughout the entire landing cycle demonstrated high consistency between the measured and simulated results, For example, the muscle activation level of BF, verified by correlation comparison, achieved an r-value of 0.985 and an RMSE value of only 0.07, indicating excellent agreement. These findings confirm the reliability of the simulated muscle activation levels (Seth et al., 2018).

### 3.2 Results of muscle synergy analysis for different single-leg landing tasks

**FIGURE 4:**
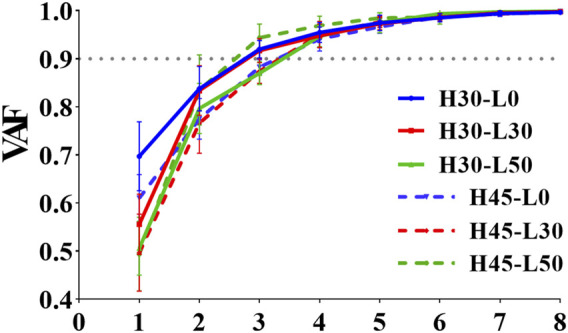
VAF values for different single-leg drop landing conditions. The figure displays the Variance Accounted For (VAF) values for different single-leg drop landings, where the solid line represents the VAF values for a 30 cm drop height, and the dashed line represents the VAF values for a 45 cm drop height. H denotes the vertical drop height (cm), and L denotes the horizontal distance (cm) of the landing position.

Figure 4 illustrates the impact of different landing heights and horizontal distances on the number, composition, and activation coefficients of muscle synergies. When subjects executed single-leg landings from a 30 cm height with horizontal distances of 0 cm and 30 cm, three consistent muscle synergies were observed.

Table 1 provides a detailed list of the muscle synergy mean VAF values and the similarity of VAF among different subjects during six single-leg landing tasks. The results showed a high consistency between different subjects (*r* > 0.9), indicating that the number of muscle synergies is consistent across participants.

**TABLE 1:** VAF values for different single leg drop landing and similarity between subjects.

Height	Weight	VAF	r
H30	L0	0.920 ± 0.018	0.988 ± 0.010
	L30	0.917 ± 0.025	0.974 ± 0.026
	L50	0.947 ± 0.022	0.986 ± 0.014
H45	L0	0.941 ± 0.025	0.978 ± 0.017
	L30	0.950 ± 0.027	0.975 ± 0.025
	L50	0.943 ± 0.028	0.967 ± 0.034

**FIGURE 5:**
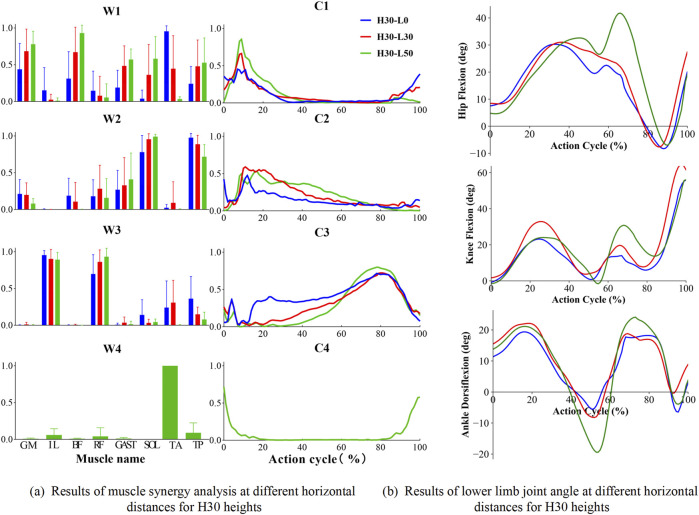
Results of muscle synergy analysis and sagittal plane angular cycle variation of lower limb joints for H30 heights. H denotes the vertical drop height (in cm), and L denotes the horizontal distance (in cm) of the landing position. **(a)** shows the muscle activation level at different horizontal lengths and 30 cm drop heights when landing on one leg; **(b)** shows the sagittal plane angle changes of hip, knee and ankle joints at different horizontal lengths and 30 cm drop heights. “W” denotes muscle synergy structure. “C” denotes the time series of muscle synergistic activation.

**FIGURE 6:**
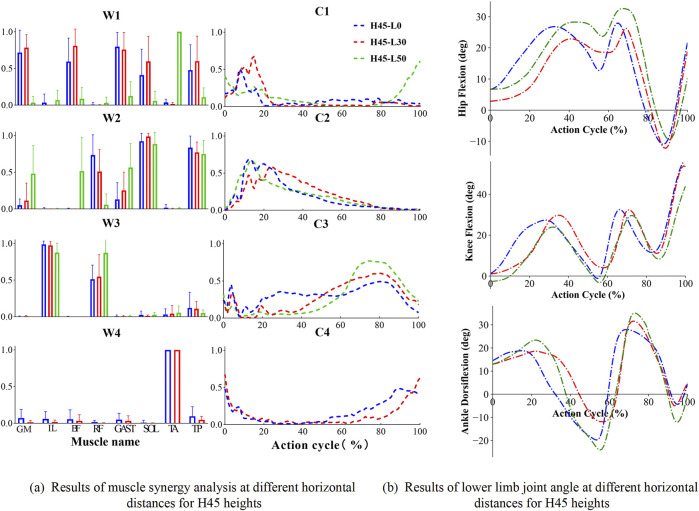
Results of muscle synergy analysis and sagittal plane angular cycle variation of lower limb joints for H45 heights.H denotes the vertical drop height (in cm), and L denotes the horizontal distance (in cm) of the landing position. **(a)** shows the muscle activation level at different horizontal lengths and 45 cm drop heights when landing on one leg; **(b)** shows the sagittal plane angle changes of hip, knee and ankle joints at different horizontal lengths and 45 cm drop heights. “W” denotes muscle synergy structure. “C” denotes the time series of muscle synergistic activation.

To investigate human neuromuscular control during single-leg landing tasks, we conducted a detailed analysis of muscle synergy weightings and activation coefficients across six different conditions (H30-L0, H30-L30, H30-L50, H45-L0, H45-L30, H45-L50). Figure 4 demonstrates the consistency of muscle synergy modules and their corresponding VAF values across different landing conditions, which serves as a quantitative foundation for understanding the muscle activation patterns visualized in Figures 5, 6.

Figure 5 illustrates that the muscle synergy structures (weighting patterns and activation coefficients) and corresponding kinematics changes in lower limb, revealing the neuromuscular control mechanisms and biomechanical adaptations during single-leg landings. For the 30 cm drop landing condition (Figure 5a), three primary synergies were identified, demonstrating how these synergies work together by modulating activation coefficients (C1, C2, C3) among key muscles. Module one predominantly activated the GM, Bi_fe, GAST, and TA mainly during the initial contact and lift-off phases. These activations supported hip extension, knee flexion, and ankle dorsiflexion, helping to manage the initial impact and prepare for subsequent propulsion. Module 2, which includes SOL and TP, primarily supported ankle plantar flexion with peak activation occurring during landing to stabilize initial contact. Lastly, Module 3, composed of IL and Rect_fem, was crucial for hip flexion and knee extension in the mid-to-late phases of landing, facilitating effective propulsion and a stable transition post-landing. As the landing height increased to 45 cm, the number of identified muscle synergy modules increased to four, indicating adaptive strategies for managing higher impact conditions. Module four is primarily composed of the TA, essential for ankle dorsiflexion at both the initial and final stages of movement. As the landing height escalated, the activation of the tibialis anterior intensified, underscoring its key role in ankle dorsiflexion. The corresponding changes in hip, knee, and ankle angles of the lower extremity during single-leg drop landings are depicted in Figure 5b. These plots represent pooled mean values across all subjects for different landing heights of 30 cm and various horizontal distances (0 cm, 30 cm, 50 cm), revealing a coordinated temporal sequence of flexion and extension across the joints. During single-leg drop landings, the hip, knee, and ankle joint angles exhibit a coordinated temporal sequence, with each joint sequentially undergoing flexion and extension to absorb landing impact and maintain body stability. Specifically, the hip joint flexion angle gradually increases during the early phase of the action cycle, peaking at 40%–70% of the cycle, and then transitions to extension, approaching a neutral position by the end of the cycle. The knee joint follows a similar trend, with its flexion angle rapidly increasing during the initial phase, peaking at 20%–40%, and then gradually extending during the mid-to-late phases, reaching near full flexion by the end of the cycle. The ankle joint initially undergoes dorsiflexion, peaking at 20%–30% of the cycle, before transitioning into plantarflexion, which reaches its maximum at 50%–60%, and finally returns to a near-neutral or slightly dorsiflexed position by the end of the cycle.

Under conditions with a horizontal distance of 50 cm, both at 30 cm and 45 cm landing heights, the number of muscle synergy modules remained three, in Figure 6a. However, there was variability in their internal weighting distribution and activation coefficients. Notably, module one showed an increased activation of ankle dorsiflexors under these conditions, highlighting the significant impact of horizontal distance on muscle synergy strategies. Similarly, similar trends in joint angles were observed as shown in Figure 6b. In addition, we note that there are some differences in some of the characteristic joint angles which were further statistically analyzed in Table 2. These joint angle patterns reflect the biomechanical adaptability of the human body under varying landing conditions, including vertical and horizontal impact forces. These muscle synergy structures and joint kinematic patterns reflect the biomechanical adaptability of the human body under varying vertical and horizontal landing conditions. The temporal coordination of lower limb joints and the activation of distinct muscle synergies highlight the neuromuscular system’s ability to efficiently absorb impact forces, maintain postural stability, and adjust to different biomechanical demands during single-leg landings.

**TABLE 2:** Maximum peak angles of hip flexion, knee flexion and ankle dorsiflexion for different single-leg drop tasks.

Maximum peak angles	Length	H30	H45	H		L		Interaction effect	
				*F*	*p*	*F*	*p*	*F*	*p*
Hip flexion	L0	30.26 ± 6.08	27.92 ± 5.40	10.20	0.001**	5.99	0.002**	1.79	0.168
	L30	31.03 ± 6.83	26.23 ± 3.45						
	L50	41.76 ± 7.48	32.58 ± 8.89						
Knee flexion	L0	55.96 ± 12.08	56.63 ± 14.62	0.10	0.753	2.92	0.054	7.42	0.000**
	L30	64.47 ± 6.71	53.59 ± 12.37						
	L50	56.07 ± 13.51	44.06 ± 16.08						
Ankle dorsiflexion	L0	19.38 ± 4.86	27.91 ± 6.75	0.79	0.375	1.58	0.206	0.09	0.912
	L30	22.11 ± 4.62	31.60 ± 7.11						
	L50	24.13 ± 6.54	35.10 ± 10.88						

“*p*” indicates significance, * denotes a p-value less than 0.05, suggesting a significant result; ** denotes a p-value less than 0.01, indicating an extremely significant result.

### 3.3 Maximum peak angles of hip flexion, knee flexion, and ankle dorsiflexion

To assess the effects of varying heights and horizontal distances on lower limb kinematics during single-leg landings, we conducted a two-way ANOVA on hip, knee, and ankle joint angles using maximum peak angles. Our findings revealed that both height (*F* = 10.202, *p* = 0.001) and distance (*F* = 5.993, *p* = 0.003) significantly influenced hip flexion, although their interaction did not (*F* = 1.787, *p* = 0.168). For knee flexion, while height alone did not have a significant effect (*F* = 0.099, *p* = 0.753), distance approached significance (*F* = 2.918, *p* = 0.055), and their interaction significantly affected knee angles (*F* = 7.423, *p* < 0.001). In contrast, ankle dorsiflexion angles were not significantly influenced by height, distance, or their interaction.

### 3.4 Similarity in muscle synergy weightings and activation coefficients

As shown in Tables 3, 4, we compared the similarity of muscle synergistic structures across participants during the single leg drop landing task. The results revealed high consistency in synergy weightings (W) among participants, particularly when four muscle synergies were identified (H30-L50, H45-L0, H45-L30), indicating that a greater number of synergies leads to higher reconstruction accuracy. Conversely, tasks with three muscle synergies (H30-L0, H30-L30, H45-L50) showed lower consistency among participants. In terms of the similarity in activation coefficients (C), although there was some consistency across synergy modules, the overall similarity in activation coefficients was slightly lower than that observed in synergy structures. Specifically, in tasks H30-L0 and H45-L0, there was relatively lower similarity in the activation coefficients of muscle synergy structures.

**TABLE 3:** Similarity of muscle co-activation timing sequences between subjects in different single-leg drop scenarios.

High	Length	C			
		Synergy1	Synergy2	Synergy3	Synergy4
H30	L0	0.397 ± 0.235	0.393 ± 0.259	0.438 ± 0.290	
	L30	0.478 ± 0.225	0.523 ± 0.254	0.507 ± 0.274	
	L50	0.675 ± 0.171	0.480 ± 0.258	0.717 ± 0.200	0.654 ± 0.222
H45	L0	0.318 ± 0.273	0.678 ± 0.189	0.315 ± 0.247	0.370 ± 0.302
	L30	0.553 ± 0.212	0.458 ± 0.272	0.440 ± 0.283	0.510 ± 0.309
	L50	0.508 ± 0.278	0.606 ± 0.223	0.617 ± 0.311	

**TABLE 4:** Similarity of muscle co-structures between subjects in different single-leg drop situations.

High	Length	W			
		Synergy1	Synergy2	Synergy3	Synergy4
H30	L0	0.491 ± 0.284	0.744 ± 0.197	0.761 ± 0.159	
	L30	0.455 ± 0.268	0.664 ± 0.262	0.777 ± 0.187	
	L50	0.801 ± 0.168	0.813 ± 0.167	0.947 ± 0.630	0.962 ± 0.045
H45	L0	0.621 ± 0.230	0.921 ± 0.118	0.930 ± 0.086	0.882 ± 0.084
	L30	0.763 ± 0.209	0.829 ± 0.157	0.904 ± 0.087	0.988 ± 0.216
	L50	0.901 ± 0.096	0.645 ± 0.286	0.938 ± 0.078	

## 4 Discussion

In this study, the kinematics, dynamics, and electromyographic signals of participants performing single-leg landing tasks at various heights and horizontal distances were analyzed using 3D motion capture systems, force platforms, and Noraxon System. The selected landing heights and horizontal distances were chosen to represent a broad spectrum of biomechanical and neuromuscular demands. Heights of 30 cm and 45 cm were used to represent distinct levels of impact load, as greater heights are associated with increased joint energy absorption and muscle activation requirements (Yeow et al., 2009; Ali et al., 2012). Similarly, horizontal distances of 0 cm, 30 cm, and 50 cm were used to assess varying levels of forward motion complexity, ranging from static to dynamic landing scenarios (Niu et al., 2011; Ali et al., 2012).

The findings confirmed that these conditions effectively captured variations in muscle synergy patterns, demonstrating the neuromuscular system’s adaptability to different task demands. The model’s accuracy in simulating muscle activation was validated by analyzing sEMG signals and comparing them with predictions from the OpenSim static optimization algorithm. VAF analysis revealed a consistent number of three muscle synergy modules under different landing conditions (H30-L0, H30-L30, H45-L50), and an increase to four modules under conditions (H30-L50, H45-L0, and H45-L30). This indicates significant effects of landing height and horizontal distance on muscle synergy patterns. Additionally, when four modules were present, there was higher consistency in synergy weights among subjects compared to the three module conditions. Although activation coefficients were more uniform between modules in the task with fewer synergies, the overall similarity in coefficients remained lower than the structural similarity. Inverse kinematics analysis further clarified the biomechanical implications of synergy patterns, showing how horizontal distance and landing height affect flexion angles of the hip, knee, and ankle joints.

The number of muscle synergies during single-leg landing tasks varies significantly with both height and horizontal distance. VAF data across different tasks show consistent muscle synergy patterns among participants, confirming the universality of muscle synergy during single-leg landing. These findings align with those of Hiroki Saito et al. (2021), who identified four muscle synergy modules for bilateral jumps and three for single leg jumps, involving key muscles like TA, RF, and GM around the hip, knee, and ankle joints. This pattern is consistent with observations by Andrea d'Avella and Emilio Bizzi in 2005 (D'Avella and Bizzi, 2005) on muscle synergy patterns in frog hind limbs during jumping, which highlighted intense activation of extensor muscles at the hip and knee joints, while the hip adductors remained inactive.

As the height and horizontal distance of single-leg landings increase, the speed of the action also accelerates. Notably, when the height reached 45 cm and the horizontal distance reached 50 cm, the fastest speed of movement was achieved, accompanied by an increase in the flexion angles of the hip, knee, and ankle joints. While some studies suggest that movement speed does not significantly affect the number of muscle synergies, others indicate that walking and running involve four and five muscle synergies respectively, suggesting that speed differences may influence synergy patterns. Moreover, as landing difficulty increases with greater height and distance, there are corresponding changes in the number of muscle synergies.

The speed of movement increases as both the height and horizontal distance of the single-leg landing increase. Notably, the fastest movement speed occurred when the height was 45 cm and the horizontal distance was 50 cm, while the flexion angles of the hip, knee, and ankle joints also increased. While some studies have shown that speed of movement has little effect on the number of muscle synergies, others suggested that walking and running involve four and five muscle synergies, respectively, implying that speed differences may influence synergy patterns. In addition, as landing height and distance increase, landing difficulty also increases, leading to changes in the number of muscle synergies. Overall, the complexity and dynamics of muscle synergy patterns adapt to changes in landing height and horizontal distance. These adaptations include not only changes in the intensity of muscle activation, but also adjustments in activation coefficients to optimize the fluidity and stability of the movement. Such adaptive synergy strategies are essential for effective movement control, energy absorption, and landing stability, demonstrating the dynamic adjustment of muscle group activation to accommodate diverse biomechanical and neuromuscular demands.

Figure 5 illustrates how the synergy patterns of eight muscles evolve with variations in height and horizontal distance during single-leg landing. Despite similarities in low-dimensional synergy modules, significant differences persist in synergy structure (W) and activation coefficients (C). Typically, the similarity in synergy structure (W) is higher than that in activation coefficients (C), indicating more accurate reconstruction of muscle synergy weights during the task. Specifically, in tasks H30-L0 and H45-L0, there was relatively lower similarity in the activation coefficients of muscle synergy structures, which might be caused by the different functional roles of the iliopsoas and rectus femoris muscles in synergistic module 3. Conversely, in task H45-L50, there was higher similarity observed in the activation coefficients across all muscle synergies.

However, there is a lower spatial consistency in muscle activation among different subjects during single-leg landing tasks, indicating variability in activation coefficients under different experimental conditions despite similar overall activation levels. Saito et al. (2018) also demonstrated that ten lower limb muscles coordinate movement through four muscle synergies during walking on horizontal and uphill treadmills, with three synergies showing similar actions and one being modulated based on specific conditions. This study finds that single-leg landing tasks are typically controlled by three main muscle synergy modules. For instance, synergy module one facilitates knee and ankle flexion upon initial ground contact and hip extension just before takeoff.

Furthermore, this study employed inverse dynamics in OpenSim to analyze how varying heights and horizontal distances affect the maximum peak angles of the hip, knee, and ankle joints during single-leg landing. It also explored the associations of these changes with muscle synergy. The results indicate that as the height of single-leg landing increases, there is a significant increase in ankle dorsiflexion and knee flexion angles. Similarly, our findings indicate that the horizontal distance had a significant effect on the maximum hip flexion angle and the maximum peak angle of hip flexion during the single-leg landings. Additionally, the landing height influenced the maximum ankle dorsiflexion angle. Moreover, both the variation of height and the horizontal distance significantly impact the maximum knee flexion angle. And greater horizontal distance leads to significant increases in hip flexion and knee flexion angles, highlighting the substantial impacts of height and horizontal distance on lower limb joint kinematics.

Analyzing muscle synergy during single-leg landings is crucial for understanding how the central nervous system plans and controls movements, revealing the body’s pre-adaptive strategies for different movements, and preventing non-contact injuries. Additionally, studying muscle coordination strategies in elite athletes can aid lower-level practitioners in enhancing their skills and improving performance. However, this study have some limitations. For instance, data collection and analysis were limited to isolated landing processes of the right leg, with no periodic tracking of the entire landing cycle, and only the landing phase was examined without addressing neuromuscular pre-contraction prior to touchdown or muscle coordination throughout the stride-to-landing process. Although our findings are based solely on healthy individuals, these preliminary data provide a valuable benchmark for understanding normal muscle synergy patterns during single-leg landing. Future research should broaden its scope to encompass the entire action cycle and investigate muscle synergy patterns across both legs, including coordination structures and activation coefficients of both the supporting and landing limbs at different movement stages. Furthermore, we acknowledge that the limited number of task repetitions in this study may influence synergy identification; hence, future investigations will specifically address the optimal number of replicates required to ensure robust and reliable extraction of muscle synergies, as suggested by previous studies (Ranaldi et al., 2023; Oliveira et al., 2014). Extending the study to include athletes, older populations, or patients with musculoskeletal impairments would be of great importance. Such investigations could employ interdisciplinary approaches—integrating biomechanics, neurophysiology, and bioinformatics—to deepen our understanding of the roles of muscle synergies in motor control and rehabilitation. This cross-disciplinary integration not only promises to refine intervention strategies for athletic training and injury prevention but also to enhance the design of personalized rehabilitation protocols, ultimately translating basic biomechanical insights into practical applications in sports science and clinical settings. Other studies have found that gender can impact exercise outcomes (Ford et al., 2006).

## 5 Conclusion

This study investigates the impact of varying heights and horizontal distances on the kinematics, kinetics, and electromyographic signals during different single-leg landing tasks using the OpenSim simulation platform. The results reveal significant variations in the number and activation coefficients of lower limb muscle synergy modules with changing task conditions. An increase in landing height and horizontal distance initially leads to an increase in synergy module count. However, beyond specific thresholds, the number of modules decreases, indicating adaptive adjustments of the neuromuscular system to increased task difficulty. Despite high similarity in synergy structures among participants, individual differences in muscle activation patterns persist. Specifically, higher landing heights lead to increased ankle dorsiflexion angles, greater horizontal distances result in increased hip flexion angles, and knee flexion angles are influenced by both factors. These findings provide a quantitative analysis of muscle synergy during single-leg landing tasks and offer new insights into the complexity of motor control through dynamic changes in muscle synergy. This is important for sports training, rehabilitation, and injury prevention, as understanding the regulatory mechanisms of muscle synergy can help design more effective training programs for athletes and rehabilitation patients, thereby enhancing performance and reducing the risk of sports-related injuries.

## Data Availability

The original contributions presented in the study are included in the article/supplementary material, further inquiries can be directed to the corresponding author.
